# High burden of birthweight-lowering genetic variants in Africans and Asians

**DOI:** 10.1186/s12916-018-1061-3

**Published:** 2018-05-24

**Authors:** Fasil Tekola-Ayele, Tsegaselassie Workalemahu, Azmeraw T. Amare

**Affiliations:** 1 Epidemiology Branch, Division of Intramural Population Health Research, Eunice Kennedy Shriver National Institute of Child Health and Human Development, National Institutes of Health, 6710B Rockledge Drive, Room 3204, Bethesda, MD 20817 USA; 2 School of Medicine, University of Adelaide, Adelaide, SA Australia

**Keywords:** Birthweight, Fetal growth, Health disparities, Multi-ancestry genetics, Genome-wide association study, Genetic risk burden, African ancestry, Asian ancestry

## Abstract

**Background:**

Birthweight is an important predictor of infant morbidity and mortality, and is associated with cardiovascular diseases, obesity, and diabetes in childhood and adulthood. Birthweight and fetal growth show regional and population variations even under similar maternal conditions, and a large proportion of these differences are not explained by environmental factors. Whether and to what extent population genetic variations at key birthweight-associated loci account for the residual birthweight disparities not explained by environmental determinants is unknown. We hypothesized that the cumulative burden of genetic variants with a birthweight-lowering effect (GRB) is different among ancestrally diverse populations.

**Methods:**

Genotype data were extracted from phase 3 of the 1000 Genomes Project for 2504 participants from 26 global populations grouped into five super-populations. GRB was calculated in offspring as the weighted sum of the number of birthweight-lowering genetic variants of 59 autosomal single-nucleotide polymorphisms associated with birthweight, and comparisons were made between Europeans and non-Europeans.

**Results:**

GRB was significantly higher in Africans (mean difference 3.15; 95% confidence interval 2.64, 3.66), admixed Americans (3.02; 2.34, 3.70), East Asians (2.85; 2.29, 3.41), and South Asians (1.07; 0.49, 1.65) compared to Europeans. Birthweight-lowering genetic variants in Africans and East Asians were enriched for rare and frequency-fixed alleles (*P* < 0.001). African and Asian populations had the greatest deviation from the expectation of the common disease-common variant hyothesis. Compared to Europeans, the GRB of ancestral alleles was significantly higher and that of derived alleles was significantly lower in non-Europeans (*P* < 0.001).

**Conclusions:**

The burden of birthweight-lowering genetic variants is higher in Africans and East Asians. This finding is consistent with the high incidence of low birthweight in the two populations. The genetic variants we studied may not be causal and the extent to which they tag the causal variants in non-Europeans is unknown; however, our findings highlight that genetic variations contribute to population differences in birthweight.

**Electronic supplementary material:**

The online version of this article (10.1186/s12916-018-1061-3) contains supplementary material, which is available to authorized users.

## Background

Birthweight is a complex multifactorial trait consistently associated with infant mortality and morbidity, with childhood obesity [1, 2], and with diseases of adulthood including type 2 diabetes, cardiometabolic diseases, and cognitive function [3–6]. There is growing interest in understanding the roles of gene–environment interactions in population differences in fetal growth following two recent studies led by the World Health Organization (WHO) [7] and the National Institute of Child Health and Human Development (NICHD) at the National Institutes of Health (NIH) [8]. The studies found regional and population differences in fetal growth, even under similar unconstrained maternal socioeconomic and nutritional conditions [7, 8]. The WHO study found significant variations in fetal growth among 12 countries from different parts of the world, and the NICHD Fetal Growth Studies found significant differences in fetal growth among four U.S. racial and ethnic populations [7, 8]. The findings in both studies corroborated earlier studies that found population differences in birthweight [9–11] and regional differences in low birthweight incidence, ranging from 6.4–7.7% in Europe and North America to 14.3% in Africa and 18.3% in Asia [12]. The WHO study also found that maternal and fetal characteristics only partially explained these differences [7], consistent with earlier observations in which established non-genetic determinants of birthweight, including socio-demographic and lifestyle-related factors, parental anthropometry, and gestational age, did not fully explain the observed birthweight differences among populations [13]. An important next step is to investigate whether and to what extent population genetic differences at key birthweight-associated loci and their interactions with environmental factors account for the residual fetal growth disparities not explained by other determinants.

To date, genome-wide association studies (GWASs) have discovered a total of 60 loci (of which 59 were autosomal) associated with birthweight [14–16]. Autosomal polymorphic single-nucleotide polymorphisms (SNPs) on the genotyping array in a recent multi-ancestry GWAS explained 15.1% of the variance in birthweight [15], reinforcing earlier heritability estimates for birthweight ranging from 25 to 31% [17, 18]. It has previously been shown that the combined effect of seven genetic loci on birthweight was similar to the effect of maternal smoking during pregnancy [16], and that of 59 loci on birthweight variance was similar to that of maternal body mass index [15], indicating a considerably high cumulative effect of the genetic loci on fetal growth. In some instances, genetic variants associated with reduced birthweight display substantial allele frequency differences among populations. For example, the rs11765649 *IGF2BP3* variant associated with lower birthweight is carried by nearly all East Asians compared to three-fourths of Europeans (99% in Han Chinese in Beijing and 74% in Utah residents with Northern and Western European Ancestry from the 1000 Genomes Project, http://www.internationalgenome.org/).

Although recent studies have illuminated the role of genetic variation on birthweight, much remains to be understood with respect to the cumulative burden of birthweight-associated loci in different populations, and to what extent they contribute to birthweight differences across populations with different ancestries. Here we tested the hypothesis that the cumulative burden of genetic variants with birthweight-lowering effect is different among ancestrally diverse human populations. Using genotype data from 26 global populations grouped into five super-populations, (1) we compared the genetic risk burden and frequency distributions of birthweight-lowering variants identified by multi-ancestry GWASs between Europeans and non-Europeans and (2) we determined whether population differences in genetic risk burden to lower birthweight vary depending on whether a variant is ancestral or derived and whether a variant is relatively benign or deleterious. Furthermore, several studies have indicated that birthweight is a strong predictor of neonatal and infant mortality [19] and optimal fetal growth and development is an important goal of pregnancy to enhance perinatal survival [20, 21]. Therefore, in global regions where low birthweight, infant mortality, socioeconomic disadvantage, and rare birthweight-lowering variants are high, it is possible that the action of negative genetic selection, which tends to wipe out deleterious birthweight-reducing variants, has been stronger to enhance the survival of offspring. Therefore, we also evaluated whether population-restricted negative selection influenced observed differences in the proportion of rare birthweight-lowering variants among populations.

## Methods

### Study population and data sets

This study included participants in phase 3 of the 1000 Genomes Project (www.1000genomes.org), which consists of 2504 individual samples from 26 global populations grouped into five super-populations: Africans (AFR, *n* = 661), admixed Americans (AMR, *n* = 347), East Asians (EAS, *n* = 504), Europeans (EUR, *n* = 503), and South Asians (SAS, *n* = 489). All participants declared themselves to be healthy at the time the samples were collected. Hence, they were very unlikely to have had severe genetic diseases during recruitment. In addition to genotype data, each participant’s sex, ethnicity, and place of origin were collected as part of the project [22].

### Selection and annotation of SNPs

We selected all 59 autosomal SNPs found to be associated with birthweight at the genome-wide level of significance in multi-ancestry GWASs involving offspring genotypes [14–16]. Given the modest effect sizes of the birthweight-associated loci, the association tests in non-Europeans did not surpass the genome-wide threshold, potentially because of the small sample sizes of the non-Europeans in the discovery study [15]. Therefore, we examined some metrics to evaluate the validity of using the loci in polygenic risk scores among diverse ancestries. The evidence supported the trans-ancestral effect of the loci on birthweight. These include:

1. A trans-ethnic meta-analysis resulted in lower *p* values compared with a European-only meta-analysis in the vast majority of loci.
2. Pooled analyses of non-Europeans and Europeans discovered seven loci (*DTL*, *HIST1H2BE*, *TRIB1*, *APOLD1*, *GPR139*, *ACTL9*, and *PEPD*) associated with birthweight, which was not achieved in the European-only cohorts.
3. The effect sizes of the SNPs were similar in both Europeans and non-Europeans, as evidenced by the strong correlations in effect sizes (*r* = 0.88; 95% confined interval CI: 0.81–0.93, *p* < 2.2 × 10^− 16^) across the 59 SNPs.
4. Heterozygosity between the trans-ancestry datasets, tested with the Q statistics, was not significant (*p* > 0.05) in 57 out of the 59 SNPs tested (the two exceptions were rs854037 in the 5q11.2 locus and rs28510415 in *PTCH1*).
5. Altogether, 50 out of 59 SNPs (85%) had directionally consistent effects in Europeans and non-Europeans.

In all, these metrics indicate that the loci have trans-ancestral effects on fetal growth.

Genotype data for the 59 SNPs were extracted from the 2504 individual samples. The SNPs included in this analysis, their birthweight-lowering alleles, nearby genes, effect size, and other annotations are reported in Additional file 1. To determine the functional and pathogenic relevance of the genetic loci, SNPs were assigned deleteriousness scores using the Combined Annotation Dependent Depletion (CADD) framework as implemented in CADD v1.2 (http://cadd.gs.washington.edu). CADD integrates functional and evolutionary importance from multiple annotation sources to generate a deleteriousness score for each genetic variant [23]. In the present analysis, the median phred-like CADD score (−10 × log10 (rank/total)) [23] was found to be 2.8. SNPs with CADD score >2.8 (*n* = 29) were considered to be relatively deleterious and SNPs with CADD score ≤2.8 (*n* = 30) were considered to be relatively benign. The ancestral or derived state of alleles for each SNP was assigned based on the Ensembl Compara 59 pipeline (six primate Enredo-Pecan-Ortheus) (http://useast.ensembl.org/).

### Statistical analyses

For each individual, the genetic risk burden for low birthweight (GRB) was calculated as the sum of the number of birthweight-lowering alleles (0, 1, or 2) per SNP multiplied by the effect size derived from the largest GWAS meta-analysis [15], followed by rescaling by the sum of the effect sizes [24]. We also generated a GRB not weighted by effect size, and no substantial differences were detected between the two metrics (Additional file 2). The mean frequencies of birthweight-lowering alleles and mean GRB were compared between Europeans and each of the four non-European populations (AFR, AMR, EAS, and SAS) with the *t*-test. The proportions of rare birthweight-lowering alleles were compared between Europeans and non-Europeans with the chi-squared test. To detect negative natural selection (purifying selection), we tested for any deviation of the allelic frequencies from the distribution expected under the neutrality model towards lower values [25]. All analyses were performed using the software program PLINK 1.9 (https://www.cog-genomics.org/plink2) [26] or R (http://www.R-project.org/).

## Results

GRB was significantly higher in Africans [mean ± standard deviation (s.d.): 64.53 ± 4.21], admixed Americans (64.41 ± 5.33), East Asians (64.23 ± 4.34), and South Asians (62.45 ± 4.59) compared to Europeans (61.38 ± 4.66) (*p* < 0.001). The direction of GRB differences between Europeans and non-Europeans varies depending on the evolutionary status of the polymorphic site (ancestral vs. derived birthweight-lowering alleles). For birthweight-lowering alleles with ancestral state (*n* = 33 SNPs), GRB was significantly higher in Africans (mean ± s.d.: 48.85 ± 3.20), admixed Americans (45.58 ± 4.01), East Asians (45.07 ± 3.06), and South Asians (43.86 ± 3.37) compared to Europeans (41.84 ± 3.36) (*p* < 0.001). In contrast, for birthweight-lowering alleles with derived state (*n* = 26 SNPs), GRB was significantly lower in Africans (mean ± s.d.: 15.68 ± 2.90), admixed Americans (18.82 ± 3.16), and South Asians (18.59 ± 3.01) compared to Europeans (19.53 ± 3.18) (*p* < 0.001). Compared to Europeans, Africans display the largest mean GRB difference of 3.15 (95% CI: 2.64, 3.66), largely driven by SNPs with ancestral birthweight-lowering alleles (mean difference 7.01; 95% CI: 6.63, 7.39) (Table 1 and Additional file 3). Further comparisons between the individual populations forming each of the super-populations revealed significant GRB differences among admixed American populations. Specifically, GRB was significantly higher in Colombians (*p* = 0.048), Mexicans (*p* = 5.2 × 10^− 5^), and Peruvians (*p* = 2.3 × 10^− 12^) compared to Puerto Ricans, and in Peruvians compared to Colombians (*p* = 0.015) (Additional files 4, 5, and 6). For each super-population, GRB was significantly higher among relatively deleterious than relatively benign loci (*p* < 0.001) and within each deleteriousness stratum, non-Europeans had higher GRB than Europeans, but the differences were not statistically significant for most comparisons (Table 2). The most deleterious birthweight-lowering variant (rs2229742 *NRIP1*) (CADD = 25.9; Additional file 1) is nearly fixed (i.e., frequency of ~100%) in Africans and East Asians, but is polymorphic in other super-populations (90.1% in EUR, 94.4% in AMR, and 94.8% in SAS).

**Table 1:** Genetic risk burden of birthweight-reducing alleles in diverse populations

Population	*N*	Mean ± s.d.	Mean difference (95% CI); *P* value
All SNPs (*n* = 59 SNPs)			
AFR	661	64.53 ± 4.21	3.15 (2.64, 3.66); <2 × 10^− 16^
AMR	347	64.40 ± 5.33	3.02 (2.34, 3.70); <2 × 10^− 16^
EAS	504	64.23 ± 4.34	2.85 (2.29, 3.41); <2 × 10^− 16^
SAS	489	62.45 ± 4.59	1.07 (0.49, 1.65); 0.0003
EUR	503	61.38 ± 4.66	ref
SNPs with ancestral birthweight-reducing alleles (*n* = 33 SNPs)			
AFR	661	48.85 ± 3.20	7.01 (6.63, 7.39); <2 × 10^− 16^
AMR	347	45.58 ± 4.01	3.74 (3.24, 4.24); <2 × 10^− 16^
EAS	504	45.07 ± 3.07	3.23 (2.83, 3.63); <2 × 10^− 16^
SAS	489	43.86 ± 3.37	2.02 (1.60, 2.44); <2 × 10^− 16^
EUR	503	41.84 ± 3.36	ref
SNPs with derived birthweight-reducing alleles (*n* = 26 SNPs)			
AFR	661	15.68 ± 2.90	−3.85 (−4.20, −3.50); <2 × 10^− 16^
AMR	347	18.82 ± 3.16	−0.70 (−1.14, −0.28); 0.0014
EAS	504	19.16 ± 3.06	−0.37 (−0.76, 0.02); 0.0602
SAS	489	18.59 ± 3.02	− 0.94 (−1.33, −0.55); 2.1 × 10^− 06^
EUR	503	19.53 ± 3.18	ref

*AFR* Africans, *AMR* admixed Americans, *CI* confidence interval, *EAS* East Asians, *EUR* Europeans, *ref* reference, *SAS* South Asians, *s.d.* standard deviation, *SNP* single-nucleotide polymorphism

**Table 2:** Genetic risk burden of birthweight-reducing alleles by deleteriousness score

Population^a^	Relatively benign (*n* = 30 SNPs)		Relatively deleterious (*n* = 29 SNPs)	
	Mean (s.d.)	Mean difference compared to EUR (95% CI); *P* value	Mean (s.d.)	Mean difference compared to EUR (95% CI); *P* value
AFR	29.85 (2.846)	1.98 (0.38, 3.58); 0.016	34.68 (3.109)	1.18 (−0.51, 2.87); 0.167
AMR	28.28 (3.449)	−0.41 (−1.35,2.17); 0.642	36.12 (3.768)	2.62 (0.76,4.48); 0.007
EAS	28.56 (3.127)	0.69 (−0.98,2.36); 0.412	35.66 (2.881)	2.16 (0.53, 3.79); 0.010
SAS	28.35 (3.332)	0.48 (−1.25,2.21); 0.579	34.10 (3.243)	0.60 (−1.12, 2.32); 0.488
EUR	27.87 (3.283)	ref	33.50 (3.360)	ref

^a^For each super-population, mean genetic risk burden of deleterious SNPs was significantly higher than that of benign SNPs. Mean differences: AFR (Africans) 4.83, AMR (admixed Americans) 7.84, EAS (East Asians) 7.1, SAS (South Asians) 5.75, EUR (Europeans) 5.63 (*p* < 0.001)

*AFR* Africans, *AMR* admixed Americans, *CI* confidence interval, *EAS* East Asians, *EUR* Europeans, *ref* reference, *SAS* South Asians, *s.d.* standard deviation, *SNP* single-nucleotide polymorphism

**Fig. 1:**
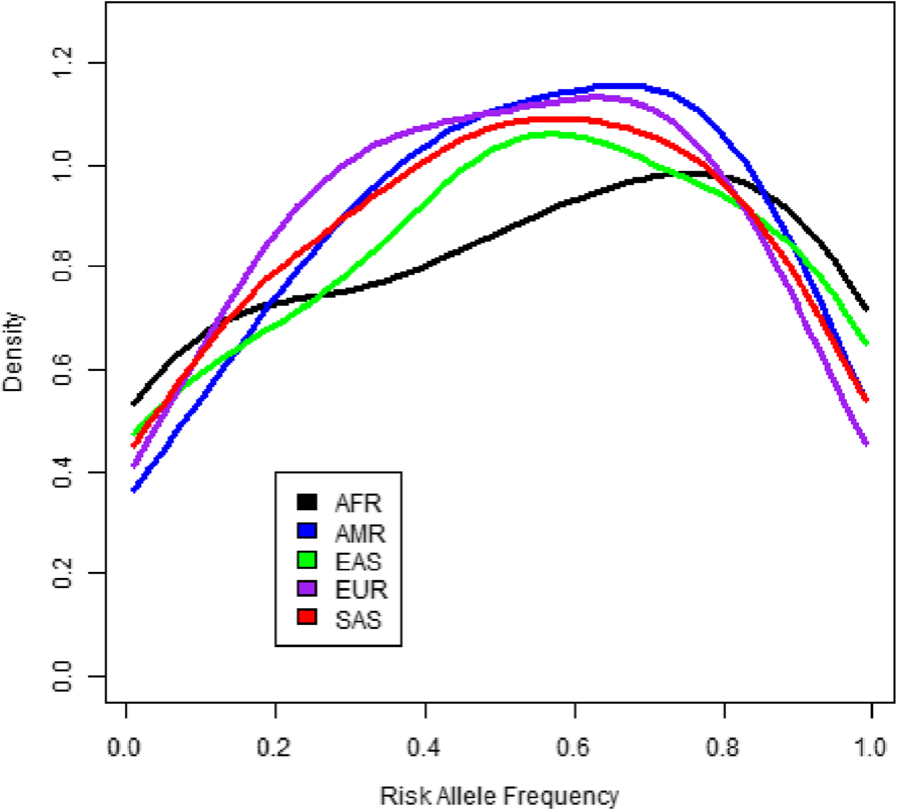
Frequency density of risk alleles associated with reduced birthweight. AFR Africans, AMR admixed Americans, EAS East Asians, EUR Europeans, SAS South Asians

**Fig. 2:**
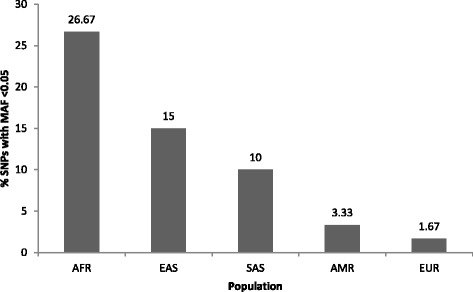
Proportion of rare SNPs (MAF < 0.05) associated with birthweight. AFR Africans, AMR admixed Americans, EAS East Asians, EUR Europeans, SAS South Asians, SNP single-nucleotide polymorphism, MAF minor allele frequency

Next, we examined population differences in allele frequency of the birthweight loci. The frequency density of the birthweight-lowering alleles was aligned with a bell shape in Europeans consistent with the expectation of the common disease-common variant hypothesis [27] but showed the greatest deviation from a bell shape in Africans and East Asians. In Europeans, the density curve peaks for birthweight-lowering alleles have a frequency of 30–40% compared to 10–20% in Africans and East Asians (Fig. 1). The proportion of rare SNPs (minor allele frequency <0.05) associated with birthweight was significantly higher in Africans (26.67%) and East Asians (15%) compared to Europeans (1.67%) (Fisher’s exact test *p* = 0.0001 and 0.0085, respectively) (Fig. 2). Moreover, of the 59 autosomal loci analyzed, five were polymorphic in Europeans but had fixed birthweight-lowering allele frequency (RAF ≥ 0.99) in non-Europeans, primarily in Africans and East Asians (rs138715366 (*YKT6-GCK*), rs11765649 (*IGF2BP3*), rs144843919 (*SUZ12P1-CRLF3*), rs2229742 (*NRIP1*), rs62240962 (*SREBF2*)). Notably, the *YKT6-GCK* locus, which had the largest birthweight-lowering effect size among the 59 loci, was fixed in each of the non-European populations (Table 3).

**Table 3:** Birthweight-reducing alleles that are polymorphic in Europeans but fixed in other populations

SNP	Gene	Chr: position (hg19)	Birthweight-reducing allele/other allele	Population in which birthweight-reducing allele is fixed (RAF > 0.99)
rs11765649	*IGF2BP3*	7: 23479013	T/C	EAS
rs138715366	*YKT6-GCK*	7: 44246271	C/T	AFR, AMR, EAS, SAS
rs144843919	*SUZ12P1-CRLF3*	17: 29037339	G/A	EAS, SAG
rs2229742	*NRIP1*	21: 16339172	G/C	AFR, EAS
rs62240962	*SREBF2*	22: 42259524	C/T	AFR

*AFR* Africans, *AMR* admixed Americans, *chr* chromosome, *EAS* East Asians, *EUR* Europeans, *RAF* risk allele frequency, *SAS* South Asians, *SNP* single-nucleotide polymorphism

To investigate whether birthweight-lowering variants were subjected to the effect of negative genetic selection that would increase the frequency of rare SNPs, we tested whether the proportion of rare birthweight-lowering alleles was higher than that of the reciprocal common birthweight-lowering alleles (RAF < 0.05 vs. RAF > 0.95; RAF < 0.5 vs. RAF > =0.5). We did not find a significantly higher proportion of rare birthweight-lowering alleles in any population (Additional file 7). Moreover, GRB was significantly higher among deleterious than benign loci in all populations (mean difference: 4.83–7.84) (Table 2), indicating that birthweight-lowering variants are not enriched for negative selection. No significant GRB differences were found between males and females.

Finally, we attempted to validate our findings using genotypes of seven global regional populations in the Human Genome Diversity Project database [28]. The median frequency of the birthweight-reducing alleles of five SNPs retrieved from the Human Genome Diversity Project database (http://spsmart.cesga.es/) was highest in Africans and the Americas (*p* = 0.028 compared to Europeans), and lowest in European and Middle Eastern populations (Additional file 8).

## Discussion

The current study found that the magnitude of the genetic burden imposed by birthweight-lowering variants is different among ancestrally diverse populations. In particular, Africans and Asians had a consistently higher burden of birthweight-lowering variants compared to Europeans. This finding is consistent with global data on the gradient of low birthweight. Regions with predominantly African and Asian ancestry populations have the highest incidence of low birthweight compared to those with predominantly Europeans ancestry populations [12]. A recent multinational study by the WHO involving healthy women with low-risk pregnancies and an unconstrained nutritional and social background from ten countries in Africa, Asia, Europe, and South America found significant differences in fetal growth across countries. The study also found significant differences in birthweight between countries. The median birthweight for countries in Africa and Asia was 400–500 g lower compared to European countries such as Norway [7]. The NICHD Fetal Growth Studies also found significant variations in fetal growth among Asian, black, Hispanic, and white ethnic groups. Asian fetuses were the smallest followed by African fetuses, and white fetuses had the largest size [8], largely consistent with the country-specific ethnic distributions in the WHO study [7].

The major determinants of these considerable variations in fetal growth and birthweight across populations remain unknown. Established maternal factors (such as maternal age, height, weight, and parity) and neonatal characteristics (such as sex) that influence fetal growth and birthweight explained only 1–2% of variations in fetal growth [7]. On the other hand, recent studies demonstrated a considerably high contribution of genetics to birthweight. There was an array-wide heritability of 15.1% [14] and a strong cumulative effect of birthweight loci that was as high as maternal smoking during pregnancy [16] and maternal body mass index [15]. Together with these observations, our findings of genetic risk burden differences among populations indicate that genetic variations and their complex interactions with environmental risk factors may contribute to observed regional and ethnic disparities in birthweight. Further, understanding these interactions may help us to understand what underlies the very slow change between 1990 and 2000 in the incidence of low birthweight in developing countries despite some improvements in their economies [12]. It may also explain why we witnessed recent decreases in birthweight in the U.S. [29, 30], with disproportionately higher declines in African-Americans than whites [31], and in Sweden [32], which could not be explained by maternal and neonatal characteristics.

In the present study, we observed population differences in the frequency spectrum of birthweight-lowering alleles. The proportion of rare risk loci was higher in individuals of African and Asian ancestry compared to those of European ancestry. The bell-shaped and symmetrical overall distribution of birthweight loci in our study has a bearing on the common disease-common variant hypothesis, which posits that common traits are most likely due to common variants with small to modest effects [27]. Nonetheless, we observed relatively higher deviations in Africans and Asians. These two findings showing differences between Europeans and Africans/Asians in the genetic variation landscape of common and rare birthweight loci may be because of population differences in the genetic architecture of birthweight and fetal growth. In addition, the overwhelming majority of GWASs, including those on birthweight, utilized samples of European ancestry populations [14–16] and most genotyping platforms are ascertained for common SNPs in European ancestry populations, limiting the power of discovery in other populations [33, 34]. These limitations may contribute to our findings of population differences in the genetic variation landscape of birthweight loci. The putative causal variants are most likely tagged by the SNPs associated with birthweight in the discovery GWAS involving European ancestry individuals; however, the extent to which those SNPs tag the causal variants in non-Europeans is not known. Therefore, we acknowledge a limitation in our study that the differences in the burden of risk alleles among populations may not represent differences in burden of causal variants. Genomic studies involving diverse population samples are warranted to discover common genetic loci associated with fetal growth and to close the gap between the estimated heritability of birthweight (25–31%) and the heritability explained by the GWAS loci discovered so far (<5%) [15, 17, 18].

In agreement with other studies [35, 36], our analysis showed a higher frequency of ancestral than derived birthweight-lowering variants in all populations, and a higher GRB of ancestral birthweight-lowering alleles in Africans and East Asians compared to Europeans. Although the well-known association of birthweight with infant mortality implied the importance of optimal birthweight to survival and reproductive fitness, our findings of (i) similar proportions of ancestral and derived birthweight-lowering alleles, (ii) higher GRB among deleterious than benign birthweight-lowering alleles, and (iii) no significantly higher proportion of rare vs. reciprocal common alleles indicate that birthweight loci were not subject to negative selection. Rather, by interrogating dbPSHP, a database of recent positive selection across human populations (http://jjwanglab.org/dbpshp), we found that 14 birthweight loci (23.3%) overlap with previously published genetic loci targeted by recent positive selection (*ZBTB7B*, *ATAD2B, CPA3, HHIP, CDKAL1, HIST1H2BE, HMGA1, SLC45A4, HHEX, NT5C2, ITPR2, CRLF3, PEPD, and SREBF2)* (Additional file 9).

## Conclusions

The present study found that non-Europeans, particularly Africans and Asians, have a higher burden of birthweight-lowering variants compared to Europeans. Moreover, the allele frequency landscape of birthweight-lowering variants in Africans and Asians has a greater deviation from the bell-shaped distribution expected under the common disease-common variant hypothesis. These findings parallel global data on the gradient of low birthweight, in which regions with predominantly African and Asian ancestry populations have the highest incidence of low birthweight and smaller fetuses that were not explained by traditional non-genetic factors. Future studies are warranted to understand the extent to which this genetic risk burden difference and its interaction with environmental factors contribute to fetal growth disparities among ancestrally diverse global populations, and to investigate the ways in which these population differences in genetic burden are governed by human demographic and adaptive history.

## Additional files

Additional file 1: SNPs included in the calculation of genetic risk burden for low birthweight. A list of the 59 autosomal SNPs associated with birthweight (Horikoshi et al., Nature Genetics, 2013. 45(1):76-U115). Additional annotations include the nearest gene, chromosome, physical position (hg19), birthweight-lowering (effect) allele and non-effect allele, effect size, whether the birthweight-reducing allele is ancestral or derived, and CADD score. (DOCX 31 kb)

Additional file 2: Comparison of weighted and unweighted genetic risk burden for low birthweight. The unweighted (**a**) and effect-size weighted (**b**) genetic risk burden (risk allele load on *y*-axis) of five super-populations is shown. (DOCX 67 kb)

Additional file 3: Genetic risk burden for low birthweight among five super-populations. The median genetic risk burden for each super-population is shown in the *y*-axis. Figures include burden for all 59 SNPs, and the ancestral and derived alleles. (DOCX 95 kb)

Additional file 4: Genetic risk burden of birthweight-lowering alleles in 26 global populations. AFR Africans, AMR admixed Americans, EAS East Asians, EUR Europeans, SAS South Asians. (DOCX 25 kb)

Additional file 5: *P* values from pairwise comparisons of genetic risk burden of birthweight-lowering alleles in 26 global populations. Colors of cells in the first row and first column indicate super-populations as indicated. *P* values <0.05 in comparison of populations belonging to the same super-population are in bold and highlighted in yellow. (DOCX 33 kb)

Additional file 6: Genetic risk burden for low birthweight among 26 global populations. Populations are shown in descending order of mean risk allele loads. Genetic risk burden of all birthweight-reducing alleles (**a**) and birthweight-reducing alleles with ancestral status (**b**) are included. (DOCX 169 kb)

Additional file 7: Frequency of rare risk alleles among populations (*n* = 59 SNPs). Of the 59 autosomal SNPs, we compared those with (i) RAF < 0.05 vs. RAF > 0.95 and (ii) RAF < 0.5 vs. RAF > =0.5, and found no significant excess of rare risk alleles in any population, indicating a lack of evidence for negative selection. (DOCX 21 kb)

Additional file 8: Published evidence for signals of recent positive selection in GWAS loci associated with birthweight. Interrogation of the http://jjwanglab.org/dbpshp database found a total of 14 birthweight GWAS loci (out of 59 autosomal loci analyzed) overlapping with previously known signals of recent positive selection. (DOCX 21 kb)

Additional file 9: Published evidence for signals of recent positive selection in GWAS loci associated with birthweight. (DOCX 23 kb)

## Abbreviations

AFR: Africans
AMR: Admixed Americans
CADD: Combined Annotation Dependent Depletion
CI: Confidence interval
EAS: East Asians
EUR: Europeans
GRB: Genetic risk burden for low birthweight
GWAS: Genome-wide association study
NICHD: *Eunice Kennedy Shriver *National Institute of Child Health and Human Development
NIH: National Institutes of Health
SAS: South Asians
s.d.: standard deviation
SNP: Single-nucleotide polymorphism
WHO: World Health Organization
