# Supplementary material for: High burden of birthweight-lowering genetic variants in Africans and Asians
Source: BMC Med. 2018 May 24;16:70. doi: 10.1186/s12916-018-1061-3 (PMC5967042; doi:10.1186/s12916-018-1061-3)
Supplement: Supplementary file 1 — SNPs included in the calculation of genetic risk burden for low birthweight. A list of the 59 autosomal SNPs associated with birthweight (Horikoshi et al., Nature Genetics, 2013. 45(1):76-U115). Additional annotations include the nearest gene, chromosome, physical position (hg19), birthweight-lowering (effect) allele and non-effect allele, effect size, whether the birthweight-reducing allele is ancestral or derived, and CADD score. (DOCX 31 kb) [file 12916_2018_1061_MOESM1_ESM.docx]

**Additional file 1: SNPs included in the calculation of genetic risk burden for low birthweight.**

| **Gene** | **SNP** | **Chr** | **Position (b37)** | **Effect Allele (Birth weight lowering)** | **Other Allele** | **Effect Size (β)** | **Exponent of β** | **Risk allele ancestral or derived** | **Phred_CADD** |
| --- | --- | --- | --- | --- | --- | --- | --- | --- | --- |
| *WNT4-ZBTB40* | rs2473248 | 1 | 22,536,643 | C | T | 0.033 | 1.03 | ancestral | 4.058 |
| *ZBTB7B* | rs3753639 | 1 | 154,986,091 | C | T | 0.031 | 1.03 | derived | 3.482 |
| *FCGR2B* | rs72480273 | 1 | 161,644,871 | C | A | 0.03 | 1.03 | derived | 2.879 |
| *near DTL* | rs61830764 | 1 | 212,289,976 | A | G | 0.022 | 1.02 | derived | 9.618 |
| *ATAD2B* | rs7575873 | 2 | 23,962,647 | A | G | 0.036 | 1.04 | ancestral | 0.572 |
| *EPAS1* | rs1374204 | 2 | 46,484,205 | T | C | 0.046 | 1.05 | ancestral | 4.473 |
| *PTH1R* | rs2242116 | 3 | 46,941,116 | A | G | 0.021 | 1.02 | derived | 3.443 |
| *ADCY5* | rs11719201 | 3 | 123,068,744 | T | C | 0.046 | 1.05 | derived | 5.389 |
| *CPA3* | rs10935733 | 3 | 148,622,968 | T | C | 0.023 | 1.02 | derived | 2.291 |
| *CCNL1-LEKR1* | rs13322435 | 3 | 156,795,468 | A | G | 0.052 | 1.05 | derived | 0.651 |
| *LCORL* | rs925098 | 4 | 17,919,811 | G | A | 0.032 | 1.03 | derived | 3.437 |
| *HHIP* | rs6537307 | 4 | 145,601,863 | G | A | 0.026 | 1.03 | ancestral | 0.82 |
| *5q11.2* | rs854037 | 5 | 57,091,783 | A | G | 0.025 | 1.03 | ancestral | 2.072 |
| *EBF1* | rs7729301 | 5 | 157,886,953 | A | G | 0.025 | 1.03 | derived | 8.879 |
| *CDKAL1* | rs35261542 | 6 | 20,675,792 | C | A | 0.044 | 1.04 | ancestral | 7.62 |
| *HIST1H2BE* | rs9379832 | 6 | 26,186,200 | A | G | 0.024 | 1.02 | ancestral | 0.028 |
| *HMGA1* | rs7742369 | 6 | 34,165,721 | G | A | 0.027 | 1.03 | ancestral | 2.483 |
| *L3MBTL3* | rs1415701 | 6 | 130,345,835 | G | A | 0.027 | 1.03 | derived | 2.937 |
| *ESR1* | rs1101081 | 6 | 152,032,917 | C | T | 0.037 | 1.04 | derived | 3.734 |
| *GNA12* | rs798489 | 7 | 2,801,803 | C | T | 0.024 | 1.02 | ancestral | 4.879 |
| *IGF2BP3* | rs11765649 | 7 | 23,479,013 | T | C | 0.026 | 1.03 | ancestral | 1.194 |
| *TBX20* | rs6959887 | 7 | 35,295,365 | A | G | 0.021 | 1.02 | derived | 2.952 |
| *YKT6-GCK* | rs138715366 | 7 | 44,246,271 | C | T | 0.244 | 1.28 | ancestral | 2.535 |
| *MLXIPL* | rs62466330 | 7 | 73,056,805 | C | T | 0.051 | 1.05 | derived | 0.403 |
| *ANK1-NKX6-3* | rs13266210 | 8 | 41,533,514 | A | G | 0.03 | 1.03 | ancestral | 0.157 |
| *TRIB1* | rs6989280 | 8 | 126,508,746 | G | A | 0.022 | 1.02 | derived | 1.261 |
| *SLC45A4* | rs12543725 | 8 | 142,247,979 | G | A | 0.022 | 1.02 | ancestral | 3.987 |
| *PTCH1* | rs28510415 | 9 | 98,245,026 | G | A | 0.053 | 1.05 | derived | 2.038 |
| *LPAR1* | rs2150052 | 9 | 113,945,067 | T | A | 0.02 | 1.02 | ancestral | 1.229 |
| *PHF19* | rs7847628 | 9 | 123,631,225 | G | A | 0.023 | 1.02 | ancestral | 8.658 |
| *STRBP* | rs700059 | 9 | 125,824,055 | G | A | 0.036 | 1.04 | ancestral | 3.015 |
| *HHEX-IDE* | rs61862780 | 10 | 94,468,643 | T | C | 0.028 | 1.03 | ancestral | 1.985 |
| *NT5C2* | rs74233809 | 10 | 104,913,940 | C | T | 0.039 | 1.04 | derived | 1.698 |
| *ADRB1* | rs7076938 | 10 | 115,789,375 | T | C | 0.035 | 1.04 | ancestral | 9.575 |
| *PLEKHA1* | rs2421016 | 10 | 124,167,512 | T | C | 0.021 | 1.02 | derived | 19.51 |
| *INS-IGF2* | rs72851023 | 11 | 2,130,620 | T | C | 0.046 | 1.05 | derived | 3.579 |
| *MTNR1B* | rs10830963 | 11 | 92,708,710 | G | C | 0.022 | 1.02 | derived | 1.411 |
| *APOLD1* | rs11055034 | 12 | 12,890,626 | C | A | 0.023 | 1.02 | ancestral | 1 |
| *ABCC9* | rs4148656 | 12 | 22,063,337 | A | G | 0.022 | 1.02 | ancestral | 2.904 |
| *ITPR2* | rs12823128 | 12 | 26,872,730 | T | C | 0.02 | 1.02 | ancestral | 1.337 |
| *HMGA2* | rs1351394 | 12 | 66,351,826 | T | C | 0.043 | 1.04 | derived | 1 |
| *IGF1* | rs7964361 | 12 | 102,994,878 | A | G | 0.038 | 1.04 | ancestral | 4.807 |
| *LINC00332* | rs2324499 | 13 | 40,662,001 | G | C | 0.023 | 1.02 | ancestral | 0.755 |
| *RB1* | rs2854355 | 13 | 48,882,363 | G | A | 0.024 | 1.02 | derived | 1.454 |
| *RNF219-AS1* | rs1819436 | 13 | 78,580,283 | C | T | 0.033 | 1.03 | ancestral | 1.716 |
| *FES* | rs12906125 | 15 | 91,427,612 | G | A | 0.023 | 1.02 | ancestral | 7.684 |
| *IGF1R* | rs7402982 | 15 | 99,193,269 | A | G | 0.023 | 1.02 | derived | 7.81 |
| *GPR139* | rs1011939 | 16 | 19,992,996 | G | A | 0.024 | 1.02 | ancestral | 0.544 |
| *CLDN7* | rs113086489 | 17 | 7,171,356 | T | C | 0.03 | 1.03 | derived | 1.834 |
| *SUZ12P1-CRLF3* | rs144843919 | 17 | 29,037,339 | G | A | 0.068 | 1.07 | ancestral | 2.919 |
| *SP6-SP2* | rs12942207 | 17 | 45,968,294 | C | T | 0.024 | 1.02 | derived | 0.968 |
| *ACTL9* | rs61154119 | 19 | 8,787,750 | T | G | 0.028 | 1.03 | ancestral | 3.714 |
| *PEPD* | rs10402712 | 19 | 33,926,013 | A | G | 0.023 | 1.02 | derived | 2.444 |
| *JAG1* | rs6040076 | 20 | 10,658,882 | C | G | 0.022 | 1.02 | ancestral | 0.337 |
| *C20orf203* | rs28530618 | 20 | 31,275,581 | A | G | 0.024 | 1.02 | ancestral | 0.095 |
| *MAFB* | rs6016377 | 20 | 39,172,728 | T | C | 0.024 | 1.02 | ancestral | 1.532 |
| *NRIP1* | rs2229742 | 21 | 16,339,172 | G | C | 0.034 | 1.03 | ancestral | 25.9 |
| *KREMEN1* | rs134594 | 22 | 29,468,456 | C | T | 0.022 | 1.02 | derived | 2.812 |
| *SREBF2* | rs62240962 | 22 | 42,259,524 | C | T | 0.047 | 1.05 | ancestral | 7.045 |
