# Supplementary material for: High burden of birthweight-lowering genetic variants in Africans and Asians
Source: BMC Med. 2018 May 24;16:70. doi: 10.1186/s12916-018-1061-3 (PMC5967042; doi:10.1186/s12916-018-1061-3)
Supplement: Supplementary file 2 — Comparison of weighted and unweighted genetic risk burden for low birthweight. The unweighted (a) and effect-size weighted (b) genetic risk burden (risk allele load on y-axis) of five super-populations is shown. (DOCX 67 kb) [file 12916_2018_1061_MOESM2_ESM.docx]

**Additional file 2: Comparison of weighted and unweighted genetic risk burden for low birthweight**

| **A** | **B** |
| --- | --- |
|  | |
