# Supplementary material for: High burden of birthweight-lowering genetic variants in Africans and Asians
Source: BMC Med. 2018 May 24;16:70. doi: 10.1186/s12916-018-1061-3 (PMC5967042; doi:10.1186/s12916-018-1061-3)
Supplement: Supplementary file 3 — Genetic risk burden for low birthweight among five super-populations. The median genetic risk burden for each super-population is shown in the y-axis. Figures include burden for all 59 SNPs, and the ancestral and derived alleles. (DOCX 95 kb) [file 12916_2018_1061_MOESM3_ESM.docx]

**Additional file 3: Genetic risk burden for low birthweight among five super-populations**
