# Supplementary material for: High burden of birthweight-lowering genetic variants in Africans and Asians
Source: BMC Med. 2018 May 24;16:70. doi: 10.1186/s12916-018-1061-3 (PMC5967042; doi:10.1186/s12916-018-1061-3)
Supplement: Supplementary file 4 — Genetic risk burden of birthweight-lowering alleles in 26 global populations. AFR Africans, AMR admixed Americans, EAS East Asians, EUR Europeans, SAS South Asians. (DOCX 25 kb) [file 12916_2018_1061_MOESM4_ESM.docx]

**Additional file 4: Genetic risk burden of birthweight lowering alleles in 26 global populations**

| **Population** | **Super population** | **Sample size** | **Birthweight reducing genetic risk burden mean (s.d.)** | **Birthweight reducing genetic risk burden mean median** |
| --- | --- | --- | --- | --- |
| ACB (African Caribbeans in Barbados)  ASW (Americans of African Ancestry in SW USA)  ESN (Esan in Nigeria)  GWD (Gambian in Western Divisions in the Gambia)  LWK (Luhya in Webuye, Kenya)  MSL (Mende in Sierra Leone)  YRI (Yoruba in Ibadan, Nigeria)  CLM (Colombians from Medellin, Colombia)  MXL (Mexican Ancestry from Los Angeles USA)  PEL (Peruvians from Lima, Peru)  PUR (Puerto Ricans from Puerto Rico)  CDX (Chinese Dai in Xishuangbanna, China)  CHB (Han Chinese in Bejing, China)  CHS (Southern Han Chinese)  JPT (Japanese in Tokyo, Japan)  KHV (Kinh in Ho Chi Minh City, Vietnam)  CEU (Utah Residents (CEPH) with Northern and Western European Ancestry)  FIN (Finnish in Finland)  GBR (British in England and Scotland)  IBS (Iberian Population in Spain)  TSI (Toscani in Italia)  BEB (Bengali from Bangladesh)  GIH (Gujarati Indian from Houston, Texas)  ITU (Indian Telugu from the UK)  PJL (Punjabi from Lahore, Pakistan)  STU (Sri Lankan Tamil from the UK) | AFR | 96 | 64.85 (4.03) | 65 |
|  | AFR | 61 | 64.33 (4.36) | 64 |
|  | AFR | 99 | 64.76 (3.72) | 64 |
|  | AFR | 113 | 63.86 (4.56) | 64 |
|  | AFR | 99 | 64.39 (4.16) | 65 |
|  | AFR | 85 | 64.88 (4.15) | 64 |
|  | AFR | 108 | 64.71 (4.45) | 65 |
|  | AMR | 94 | 64.24 (5.15) | 64 |
|  | AMR | 64 | 65.78 (4.88) | 66 |
|  | AMR | 85 | 67.19 (4.29) | 67 |
|  | AMR | 104 | 61.43 (5.05) | 62 |
|  | EAS | 93 | 63.55 (4.36) | 64 |
|  | EAS | 103 | 64.96 (4.36) | 64 |
|  | EAS | 105 | 64.11 (3.88) | 64 |
|  | EAS | 104 | 65.18 (4.05) | 65 |
|  | EAS | 99 | 63.22 (4.78) | 63 |
|  | EUR | 99 | 61.49 (4.80) | 62 |
|  | EUR | 99 | 61.43 (4.66) | 62 |
|  | EUR | 91 | 62.22 (4.62) | 62 |
|  | EUR | 107 | 60.80 (4.62) | 61 |
|  | EUR | 107 | 61.07 (4.57) | 61 |
|  | SAS | 86 | 61.58 (4.40) | 62 |
|  | SAS | 103 | 62.17 (4.97) | 62 |
|  | SAS | 102 | 62.22 (4.03) | 62 |
|  | SAS | 96 | 62.38 (4.54) | 62 |
|  | SAS | 102 | 63.79 (4.73) | 64 |
