# Supplementary material for: High burden of birthweight-lowering genetic variants in Africans and Asians
Source: BMC Med. 2018 May 24;16:70. doi: 10.1186/s12916-018-1061-3 (PMC5967042; doi:10.1186/s12916-018-1061-3)
Supplement: Supplementary file 5 — P values from pairwise comparisons of genetic risk burden of birthweight-lowering alleles in 26 global populations. Colors of cells in the first row and first column indicate super-populations as indicated. P values <0.05 in comparison of populations belonging to the same super-population are in bold and highlighted in yellow. (DOCX 33 kb) [file 12916_2018_1061_MOESM5_ESM.docx]

**Additional file 5: P-values from pairwise comparisons of genetic risk burden of birthweight lowering alleles in 26 global populations**

|  | ACB | ASW | BEB | CDX | CEU | CHB | CHS | CLM | ESN | FIN | GBR | GIH | GWD | IBS | ITU | JPT | KHV | LWK | MSL | MXL | PEL | PJL | PUR | STU | TSI |
| --- | --- | --- | --- | --- | --- | --- | --- | --- | --- | --- | --- | --- | --- | --- | --- | --- | --- | --- | --- | --- | --- | --- | --- | --- | --- |
| ASW | 1.0000 |  |  |  |  |  |  |  |  |  |  |  |  |  |  |  |  |  |  |  |  |  |  |  |  |
| BEB | 0.00017 | 0.08642 |  |  |  |  |  |  |  |  |  |  |  |  |  |  |  |  |  |  |  |  |  |  |  |
| CDX | 1.0000 | 1.00000 | 0.99964 |  |  |  |  |  |  |  |  |  |  |  |  |  |  |  |  |  |  |  |  |  |  |
| CEU | 0.00011 | 0.06131 | 1.00000 | 0.71400 |  |  |  |  |  |  |  |  |  |  |  |  |  |  |  |  |  |  |  |  |  |
| CHB | 1.0000 | 1.00000 | 0.00012 | 1.00000 | 7.3e05 |  |  |  |  |  |  |  |  |  |  |  |  |  |  |  |  |  |  |  |  |
| CHS | 1.0000 | 1.00000 | 0.01538 | 1.00000 | 0.01001 | 1.00000 |  |  |  |  |  |  |  |  |  |  |  |  |  |  |  |  |  |  |  |
| CLM | 1.0000 | 1.00000 | 0.08032 | 1.00000 | 0.05621 | 1.00000 | 1.00000 |  |  |  |  |  |  |  |  |  |  |  |  |  |  |  |  |  |  |
| ESN | 1.0000 | 1.00000 | 0.00014 | 1.00000 | 8.7e05 | 1.00000 | 1.00000 | 1.00000 |  |  |  |  |  |  |  |  |  |  |  |  |  |  |  |  |  |
| FIN | 0.000042 | 0.03787 | 1.00000 | 0.44762 | 1.00000 | 2.9e05 | 0.00470 | 0.03333 | 3.2e05 |  |  |  |  |  |  |  |  |  |  |  |  |  |  |  |  |
| GBR | 0.01711 | 1.00000 | 1.00000 | 1.00000 | 1.00000 | 0.01164 | 0.77603 | 1.00000 | 0.01725 | 1.00000 |  |  |  |  |  |  |  |  |  |  |  |  |  |  |  |
| GIH | 0.01298 | 1.00000 | 1.00000 | 1.00000 | 1.00000 | 0.00883 | 0.61401 | 1.00000 | 0.01297 | 1.00000 | 1.00000 |  |  |  |  |  |  |  |  |  |  |  |  |  |  |
| GWD | 1.0000 | 1.00000 | 0.15305 | 1.00000 | 0.10484 | 1.00000 | 1.00000 | 1.00000 | 1.00000 | 0.05838 | 1.00000 | 1.00000 |  |  |  |  |  |  |  |  |  |  |  |  |  |
| IBS | 0.00000008 | 0.00080 | 1.00000 | 0.00813 | 1.00000 | 5.9e08 | 1.7e05 | 0.00051 | 4.2e08 | 1.00000 | 1.00000 | 1.00000 | 0.00053 |  |  |  |  |  |  |  |  |  |  |  |  |
| ITU | 0.00242 | 0.84078 | 1.00000 | 1.00000 | 1.00000 | 0.00166 | 0.21852 | 0.84382 | 0.00199 | 1.00000 | 1.00000 | 1.00000 | 1.00000 | 1.00000 |  |  |  |  |  |  |  |  |  |  |  |
| JPT | 1.0000 | 1.00000 | 8.9e06 | 1.00000 | 5.3e06 | 1.00000 | 1.00000 | 1.00000 | 1.00000 | 1.8e06 | 0.00148 | 0.00109 | 1.00000 | 1.7e09 | 0.00011 |  |  |  |  |  |  |  |  |  |  |
| KHV | 1.0000 | 1.00000 | 1.00000 | 1.00000 | 1.00000 | 1.00000 | 1.00000 | 1.00000 | 1.00000 | 1.00000 | 1.00000 | 1.00000 | 1.00000 | 0.09518 | 1.00000 | 0.62970 |  |  |  |  |  |  |  |  |  |
| LWK | 1.0000 | 1.00000 | 0.00492 | 1.00000 | 0.00319 | 1.00000 | 1.00000 | 1.00000 | 1.00000 | 0.00149 | 0.27039 | 0.21249 | 1.00000 | 5.7e06 | 0.06948 | 1.00000 | 1.00000 |  |  |  |  |  |  |  |  |
| MSL | 1.0000 | 1.00000 | 0.00037 | 1.00000 | 0.00023 | 1.00000 | 1.00000 | 1.00000 | 1.00000 | 0.00010 | 0.02731 | 0.02103 | 1.00000 | 3.3e07 | 0.00521 | 1.00000 | 1.00000 | 1.00000 |  |  |  |  |  |  |  |
| MXL | 1.0000 | 1.00000 | 8.9e05 | 1.00000 | 5.9e05 | 1.00000 | 1.00000 | 1.00000 | 1.00000 | 3.2e05 | 0.00365 | 0.00288 | 1.00000 | 3.7e07 | 0.00108 | 1.00000 | 0.41440 | 1.00000 | 1.00000 |  |  |  |  |  |  |
| PEL | 0.07581 | 0.04385 | 4.4e12 | 2.5e05 | 2.2e12 | 0.17776 | 0.00026 | **0.01548** | 0.02329 | 5.7e13 | 1.8e09 | 1.2e09 | 0.00013 | 2.6e16 | 2.6e11 | 0.40897 | 4.8e06 | 0.00453 | 0.15432 | 1.00000 |  |  |  |  |  |
| PJL | 0.02968 | 1.00000 | 1.00000 | 1.00000 | 1.00000 | 0.02018 | 1.00000 | 1.00000 | 0.02991 | 1.00000 | 1.00000 | 1.00000 | 1.00000 | 1.00000 | 1.00000 | 0.00252 | 1.00000 | 0.46504 | 0.04726 | 0.00619 | 2.5e09 |  |  |  |  |
| PUR | 0.000097 | 0.05245 | 1.00000 | 0.60875 | 1.00000 | 6.7e05 | 0.00881 | **0.04816** | 8.1e05 | 1.00000 | 1.00000 | 1.00000 | 0.09006 | 1.00000 | 1.00000 | 5.0e06 | 1.00000 | 0.00282 | 0.00021 | **5.2e05** | **2.3e12** | 1.00000 |  |  |  |
| STU | 1.0000 | 1.00000 | 0.35178 | 1.00000 | 0.24735 | 1.00000 | 1.00000 | 1.00000 | 1.00000 | 0.14864 | 1.00000 | 1.00000 | 1.00000 | 0.00219 | 1.00000 | 1.00000 | 1.00000 | 1.00000 | 1.00000 | 1.00000 | 0.00022 | 1.00000 | 0.21138 |  |  |
| TSI | 0.0000007 | 0.00349 | 1.00000 | 0.03891 | 1.00000 | 5.0e07 | 0.00013 | 0.00251 | 4.0e07 | 1.00000 | 1.00000 | 1.00000 | 0.00314 | 1.00000 | 1.00000 | 1.7e08 | 0.36817 | 4.2e05 | 2.5e06 | 1.9e06 | 2.7e15 | 1.00000 | 1.00000 | 0.01109 |  |
| YRI | 1.0000 | 1.00000 | 0.00067 | 1.00000 | 0.00043 | 1.00000 | 1.00000 | 1.00000 | 1.00000 | 0.00018 | 0.05108 | 0.03939 | 1.00000 | 4.9e07 | 0.00966 | 1.00000 | 1.00000 | 1.00000 | 1.00000 | 1.00000 | 0.04099 | 0.08859 | 0.00038 | 1.00000 | 4.0e06 |

| African: |  |  | Admixed American: |  | East Asian: |  | European: |  |  | South Asian: |  |
| --- | --- | --- | --- | --- | --- | --- | --- | --- | --- | --- | --- |
