# Supplementary material for: High burden of birthweight-lowering genetic variants in Africans and Asians
Source: BMC Med. 2018 May 24;16:70. doi: 10.1186/s12916-018-1061-3 (PMC5967042; doi:10.1186/s12916-018-1061-3)
Supplement: Supplementary file 6 — Genetic risk burden for low birthweight among 26 global populations. Populations are shown in descending order of mean risk allele loads. Genetic risk burden of all birthweight-reducing alleles (a) and birthweight-reducing alleles with ancestral status (b) are included. (DOCX 169 kb) [file 12916_2018_1061_MOESM6_ESM.docx]

**Additional file 6: Genetic risk burden for low birthweight among 26 global populations**

|  |
| --- |
|  |
|  |
|  |
