# Supplementary material for: High burden of birthweight-lowering genetic variants in Africans and Asians
Source: BMC Med. 2018 May 24;16:70. doi: 10.1186/s12916-018-1061-3 (PMC5967042; doi:10.1186/s12916-018-1061-3)
Supplement: Supplementary file 7 — Frequency of rare risk alleles among populations (n = 59 SNPs). Of the 59 autosomal SNPs, we compared those with (i) RAF < 0.05 vs. RAF > 0.95 and (ii) RAF < 0.5 vs. RAF > =0.5, and found no significant excess of rare risk alleles in any population, indicating a lack of evidence for negative selection. (DOCX 21 kb) [file 12916_2018_1061_MOESM7_ESM.docx]

**Additional file 7: Frequency of rare risk alleles among populations (n=59 SNPs)**

| Super-population | Rare SNPs | | Minor vs. major alleles | |
| --- | --- | --- | --- | --- |
|  | RAF <0.05  # (%) | RAF >0.95  # (%) | RAF<0.5  #(%) | RAF>=0.5  #(%) |
| AFR | 5 (8.47) | 11 (18.64) | 5 (8.47) | 54 (91.53) |
| AMR | 1 (1.69) | 1 (1.69) | 1 (1.69) | 58 (98.31) |
| EAS | 4 (6.78) | 5 (8.47) | 4 (6.78) | 55 (93.22) |
| EUR | 0 (0) | 1 (1.69) | 0 (0) | 59 (100.0) |
| SAS | 3 (5.08) | 3 (5.08) | 3 (5.08) | 56 (94.92) |
