# Supplementary material for: High burden of birthweight-lowering genetic variants in Africans and Asians
Source: BMC Med. 2018 May 24;16:70. doi: 10.1186/s12916-018-1061-3 (PMC5967042; doi:10.1186/s12916-018-1061-3)
Supplement: Supplementary file 8 — Published evidence for signals of recent positive selection in GWAS loci associated with birthweight. Interrogation of the http://jjwanglab.org/dbpshp database found a total of 14 birthweight GWAS loci (out of 59 autosomal loci analyzed) overlapping with previously known signals of recent positive selection. (DOCX 21 kb) [file 12916_2018_1061_MOESM8_ESM.docx]

**Additional file 8: Distribution of birthweight lowering alleles found in the HGDP dataset**

| **Population group** | **rs6537307 G**  **mean** | **rs7742369 G**  **mean** | **rs13266210 A**  **mean** | **rs2421016 A**  **mean** | **rs11055034 C**  **mean** | **Overall median (IQR)** |
| --- | --- | --- | --- | --- | --- | --- |
| **Africa** | 0.132 | 0.603 | 0.779 | 0.196 | 0.918 | 0.603 (0.196-0.779) |
| **America** | 0.492 | 0.766 | 0.867 | 0.852 | 0.914 | 0.852 (0.766-0.867)* |
| **Europe** | 0.440 | 0.171 | 0.734 | 0.449 | 0.708 | 0.449 (0.441-0.708) |
| **Middle East** | 0.420 | 0.209 | 0.724 | 0.445 | 0.742 | 0.445 (0.42-0.724) |
| **Central-South Asia** | 0.525 | 0.150 | 0.818 | 0.520 | 0.728 | 0.525 (0.52-0.728) |
| **Oceania** | 0.018 | 0.268 | 1.000 | 0.679 | 0.885 | 0.679 (0.268-0.885) |
| **East Asia** | 0.231 | 0.146 | 0.884 | 0.638 | 0.560 | 0.56 (0.231-0.638) |
| *Median differences comparing the population with Europeans are statistically significant (Wilcoxon rank sum test W = 23, p=0.03). | | | | | | |
