# Supplementary material for: High burden of birthweight-lowering genetic variants in Africans and Asians
Source: BMC Med. 2018 May 24;16:70. doi: 10.1186/s12916-018-1061-3 (PMC5967042; doi:10.1186/s12916-018-1061-3)
Supplement: Supplementary file 9 — Published evidence for signals of recent positive selection in GWAS loci associated with birthweight. (DOCX 23 kb) [file 12916_2018_1061_MOESM9_ESM.docx]

**Additional file 9: Published evidence for signals of recent positive selection in GWAS loci associated with birthweight**

| Birth weight GWAS | | | Overlapping signals of recent positive selection | | |
| --- | --- | --- | --- | --- | --- |
| **Birthweight GAWS gene** | **Birthweight GAWS SNP** | **Birthweight GAWS SNP position** | **Population** | **locus** | **PubMed ID** |
| *ZBTB7B* | rs3753639 | 154986091 | Bangladeshi | chr1:154931708-155040297 | 23825302 |
|  | rs3753639 | 154986091 | CHB | chr1:154810683-155868625 | 23731540 |
|  | rs3753639 | 154986091 | JPT | chr1:154811127-154986793 | 23731540 |
|  | rs3753639 | 154986091 | Hazara-Xibo-Japanese-Naxi-Han | chr1:154801247-155086148 | 19924308 |
| *ATAD2B* | rs7575873 | 23962647 | CHB | chr2:23885168-24632122 | 23731540 |
| *CPA3* | rs10935733 | 148622968 | CHM-YRI | chr3:148606725-148867988 | 19424418 |
| *HHIP* | rs6537307 | 145601863 | Brahui | chr4:145203531-145633530 | 19924308 |
| *CDKAL1* | rs35261542 | 20675792 | ASN-CEU | Chr6: 20534687- 21232634 | 21188420 |
| *HIST1H2BE* | rs9379832 | 26186200 | JPT | chr6:26124303-26554968 | 23731540 |
| *HMGA1* | rs7742369 | 34165721 | Bangladeshi | chr6:34144866-34275931 | 23825302 |
|  | rs7742369 | 34165721 | MEX | chr6:34078463-36127469 | 23731540 |
| *SLC45A4* | rs12543725 | 142247979 | CEU | chr8:142130818-142330818 | 19844263 |
| *HHEX* | rs61862780 | 94468643 | JPT | chr10:92957222-95395727 | 23731540 |
|  | rs61862780 | 94468643 | Central Asian | rs1111875 | 23340510 |
|  | rs61862780 | 94468643 | ASN-CEU | Chr10: 94449680-94455408 | 21188420 |
| *NT5C2* | rs74233809 | 104913940 | YRI | Chr10:104847773-104953063 | 20416085 |
|  | rs74233809 | 104913940 | CEU-YRI-CHB | Chr10:104847773-104953063 | 16371466 |
| *ITPR2* | rs12823128 | 26872730 | Maya | chr12:26297832-26872730 | 19924308 |
| *CRLF3* | rs144843919 | 29037339 | CHB | chr17:28550814-29395746 | 23731540 |
|  | rs144843919 | 29037339 | CHD | chr17:28552986-29395746 | 23731540 |
| *PEPD* | rs10402712 | 33926013 | GDP | chr19:33889369-34061112 | 18524786 |
| *SREBF2* | rs62240962 | 42259524 | She-Dai-Papuan | chr22:42126819-42873532 | 19924308 |
